# Case Report: Microsurgical excision of grade 5 cerebral AVM

**DOI:** 10.12688/f1000research.7257.2

**Published:** 2016-07-04

**Authors:** Sunil Munakomi, Binod Bhattarai, Iype Cherian

**Affiliations:** 1 College of Medical Sciences, Bharatpur, Chitwan, 44207, Nepal

**Keywords:** arteriovenous malformation, AVM, grading, management

## Abstract

In this case report, we discuss the microsurgical management of a Spetzler-Martin grade 5 arteriovenous malformation (AVM) in a young boy who presented with a hemorrhagic episode and had a high calculated risk of rebleeding. We also outline the rationale for choosing the management option.

## Introduction

As of now, many tenets exist regarding management of high grade cerebral arterio-venous malformation (AVM) management, making a rigid algorithm impossible to create. In experienced hands, microsurgery proved to have better results, compared to other treatments ^1, 2^. Herein, we report a microsurgical management of a grade 5 arteriovenous malformation (AVM) in a young patient with a high predicted risk for rebleeding.

## Case report

**Figure 1.**
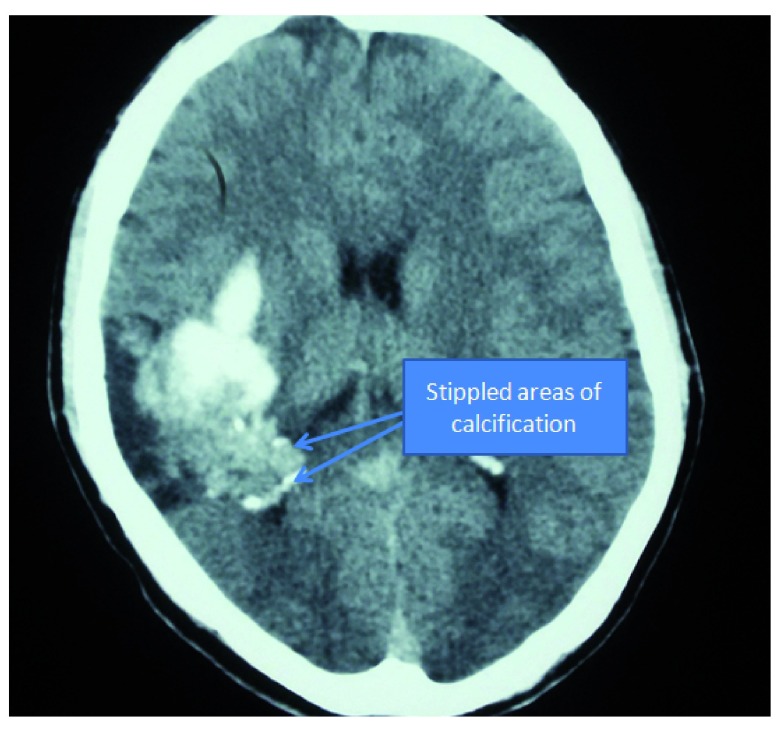
CT showing presence of hyperdense lesion in the right parietal region with stippled calcification on the periphery.

**Figure 2.**
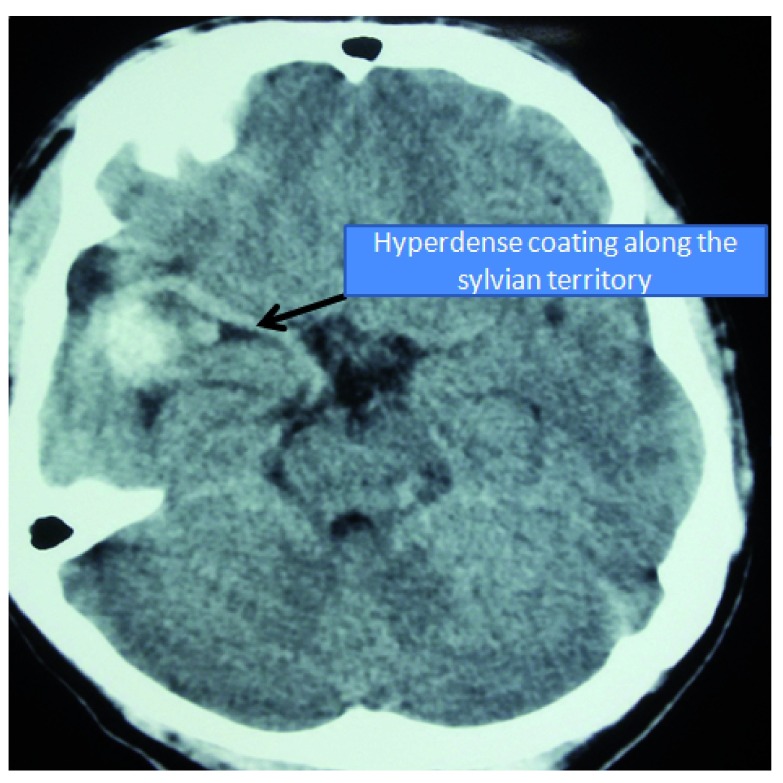
CT showing presence of coating along the MCA territory.

**Figure 3.**
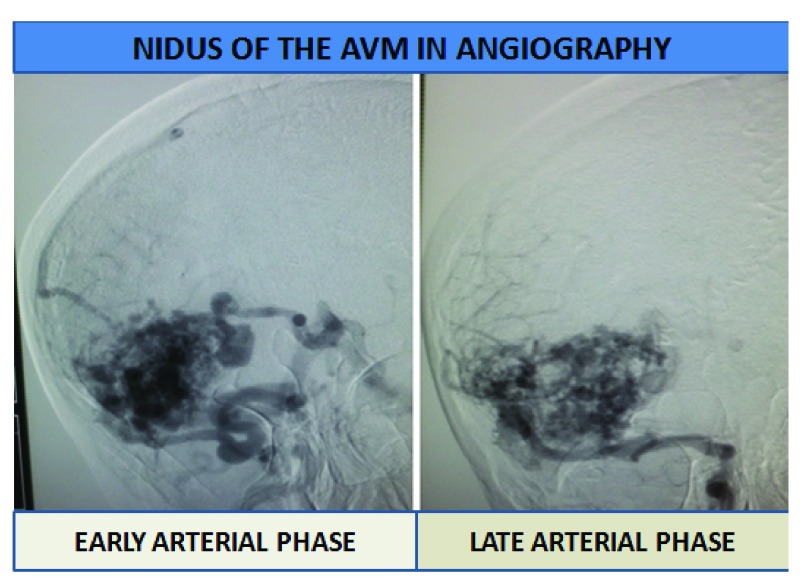
Angiogram showing the nidus of the AVM during angiography.

**Figure 4.**
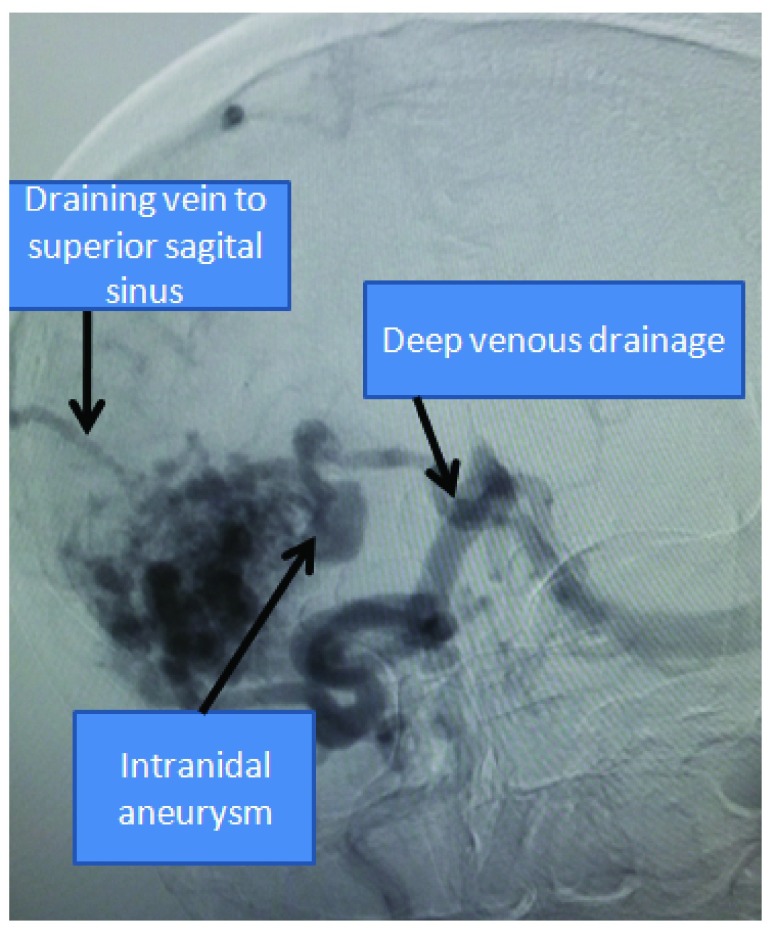
Angiogram showing the major deep draining vein and a single vein to the superior sagittal sinus.

A 22-year-old Brahmin male from Khaireni, a remote village in Nepal, presented to our emergency room with a sudden-onset severe headaches and left-sided weakness over the last 24 hours. Physical examination revealed a Glasgow coma scale (GCS) of 14/15 with left-sided hemiparesis of 3+/5(Medical research council grading). Medical history was significant for a few episodes of paroxysmal headaches since last couple of years, which improved after taking 500 mg Paracetamol tablet on an ‘as needed’ basis. The frequency and intensity of the headache had worsened in the last few months. There was no significant family history. An urgent head computerized tomogram (CT) revealed evidence of a hyperdense lesion with peripheral stippled calcification on the right side in the territory of posterior limb of internal capsule and the retro-thalamic region ( Figure 1). There was also coating of vessel along the middle cerebral artery (MCA) territory ( Figure 2) and hyperdensity along the deep venous territory. A four-vessel diagnostic carotid angiography revealed Grade 5 Spetzler-Martin AVM in the right sub corticol region with feeders from lenticulostiates of the middle cerebral artery ( Figure 3). Drainage was to the deep draining veins and also to the superior sagittal sinus ( Figure 4).

**Figure 5.**
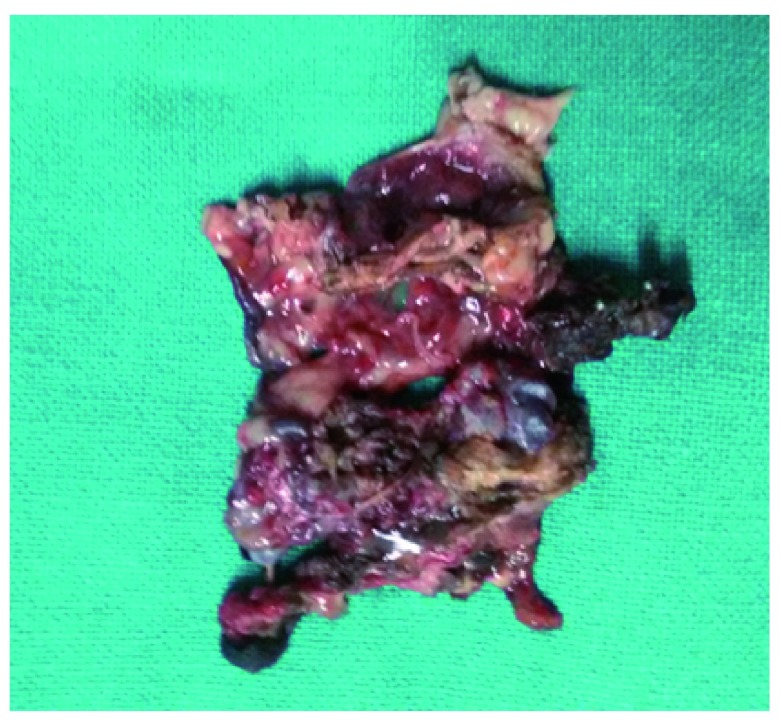
Excised AVM nidus.

Multiple factors such as young age at presentation, the fact that the lesion had bled, presentation of patient with deficits associated with the lesion on the non-dominant side, presence of deep venous drainage and intra-nidal aneurysm led to a high calculated risk for rebleeding in the patient. We therefore decided on surgical management, despite the high grade of the lesion. After explanation of the risks of the treatment and role of adjuvants in the form of radiosurgery and embolisation the patient was taken up for microsurgical excision. Since the facility of radiosurgery is not available in the country, we only had the option of embolisation of the feeders prior to the surgical excision of the lesion. However, since the lesion had only low velocity feeders from the lenticulostriate vessels, we opted for direct microsurgical management. After a liberal craniotomy, basal cisterns were opened to gain access to the M1 branch of the MCA. We identified the major deep draining vein that was looping over the MCA bifurcation with the help of Indocyanine Green (ICG) venography. We placed a temporary clip on M1, then made a minimal corticostomy over the parietal cortex and continued our dissection over the gliotic tissue surrounding the AVM taking care of the minimal bleeders with the help of bipolar cauterization and avoiding inadvertent entry to the nidus. Lastly a clip was applied to the draining vein after completely dissecting the AVM nidus. The lesion was finally excised ( Figure 5). Complete hemostasis was confirmed.

**Figure 6.**
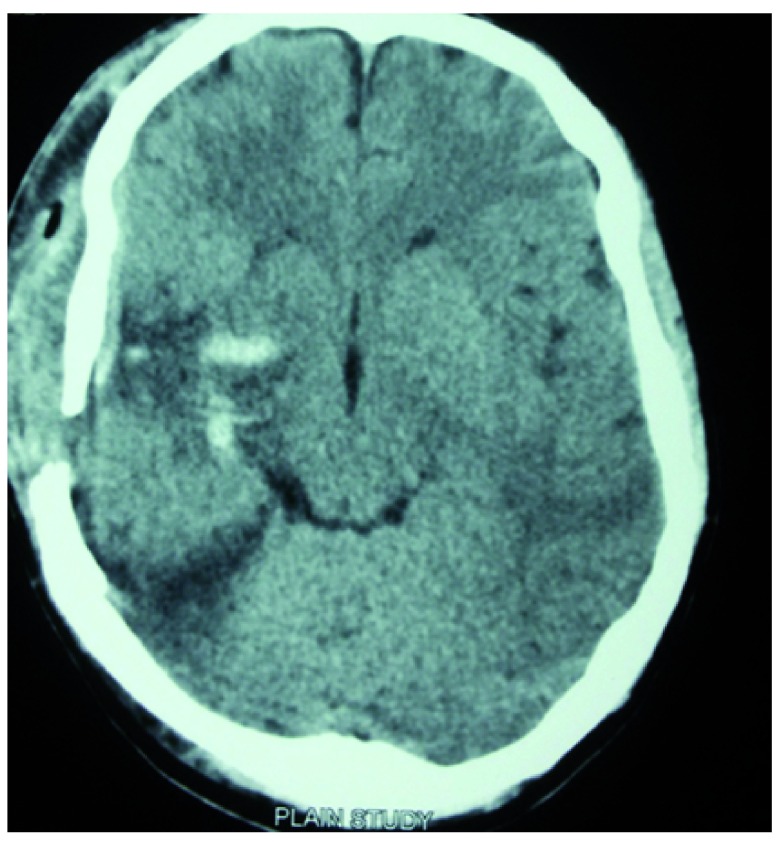
Post-operative scan with no haemorrhage or the evidence of vasospasm.

**Figure 7.**
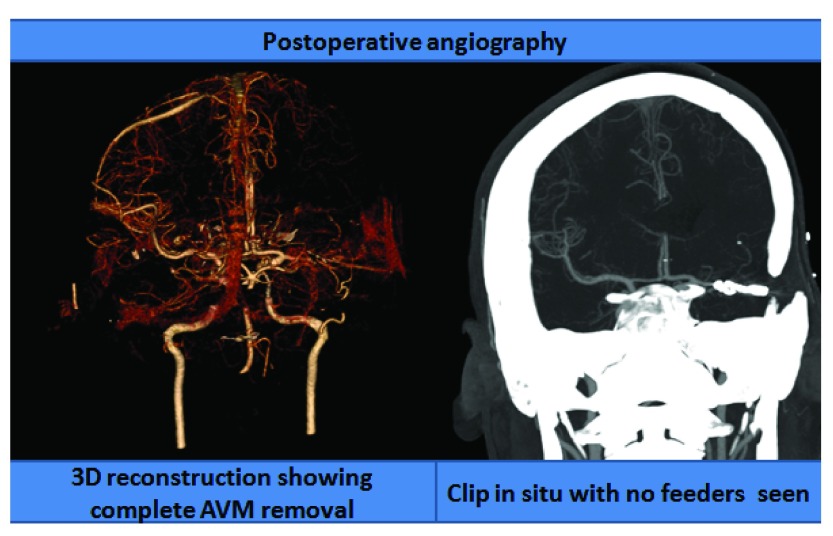
Post operative angiography revealing complete excision of the AVM with no residual feeders left.

Postoperatively his blood pressure was rigorously monitored so as not to overshoot the mean arterial pressure above 100 mm of mercury so as to prevent breakthrough perfusion rebleeding. Patient was started on Sodium Valproate (1 gm stat followed by 300 mg IV 8 hourly) and Nimodipine (60 mg 4 hourly via nasogastric tube) for seizure and vasospasm prophylaxis, respectively. Repeat head CT scan the following morning revealed no cavity hematoma or any evidence of vasospasm ( Figure 6). Patient was extubated uneventfully. He had hemiparesis of 3+ in upper limbs and 3 in lower limbs. Patient was started on physiotherapy and finally discharged home on the 7 ^TH^ post-operative day after removal of sutures. Patient came for follow-up 2 weeks later walking on his own with left upper limb weakness of grade 3+/5. The Nimodipine was tapered off in the subsequent three weeks. The patient was advised to continue Na Valproate 300 mg orally three times a day for at least a year. Post operative angiography revealed complete excision of the AVM with no remaining feeders ( Figure 7). The patient followed up in the outpatient clinic 6 months later with minimal pronator drift on the left arm and grade 2 spasticity on his left leg.

## Discussion

Bleeding within the AVM is considered a significant predictor of rebleeding. Other important factors moderating risk of rebleeding include deep venous drainage ^3^. Studies have verified that the risk of rebleeding under these circumstances is as high as 34.4% compared to just 0.9% per year in patients without these risk factors ^4, 5^. Another important factor to be considered while calculating the risk of rebleeding is the presence of concurrent aneurysm within the AVM (6.93% with aneurysm Vs 3.99% without aneurysm) ^3^.

Up to 40% of cases with AVM manifest neurological deficits ^6^, mostly attributable to hemorrhage. A minority of only 5% to 15% of such deficits are related to factors such as coronary steal phenomenon and venous hypertension ^7– 9^.

The Spetzler-Martin Scale is used to estimate the risk of surgical resection of an AVM with higher grades being associated with greater surgical morbidity and mortality ^10^. Multivariate studies have shown this grading system to reliably predict permanent major morbidity or mortality at the following levels: Grade I (4%), Grade II (10%), Grade III (18%), Grade IV (31%), and Grade V (37%) ^11^. This data has been further validated prospectively, and this grading system remains the most widely used among neurosurgeons and neurointerventionalists ^12^.

Han *et al.* reported the management of 73 grade 4 and 5 lesions and found the annual hemorrhage rate for untreated lesions to be only 1.5% versus 10.4% for partially treated lesions ^13^. Grade IV or V lesions are only treated in circumstances of progressive neurological deterioration from hemorrhage, vascular steal, or seizure as seen in our case, which had a high risk of rebleeding because of presentation at young age with hemorrhagic episode, large size of the nidus, deep venous drainage pattern and associated aneurysm within the AVM.

There is time-lag of about two years following radiosurgery for complete obliteration of the nidus in the lesion. The risk of hemorrhage in this time period is around 4.8% per year ^14^ which parallels the natural history of the lesion after bleeding. However there is a risk of inadvertent radiation injury to the adjacent eloquent brain area ^15^ and also a risk of symptomatic radiation necrosis in around 9% of cases ^15, 16^.

The main indication for other embolisation options in such a high grade of AVM is in order to downgrade the lesion and to minimise the intraoperative blood loss so as to make the lesion amenable for microsurgical excision, which bears an acceptable complication rate of around just 6.5% ^17^. One study has shown that the deep venous drainage, higher grade of the lesions and the periprocedural hemorrhage are predictors of post procedural complications following the embolisation treatment ^17^.

In our case there were only few feeders from the lenticulostriate branches from MCA: not ideal for embolization. Partial embolisation of the lesion will not reduce the risk of hemorrhage to zero ^3^. Partial embolisation of the high grade lesions are only justified in few circumstances, such as in vascular steal phenomenon or an AVM with associated aneurysm ^18^.

## Conclusion

In a few selected cases who have a high calculated risk of rebleeding, microsurgical excision remains a therapeutic option even for a high grade AVM especially in centers with limited resources for intervention and radiosurgery. However, all the patients should be well counseled about the available alternative mode of intervention and the associated risks. The management plan in each patient should be tailored addressing factors such as age of the patient, mode of presentation, grade of the lesion, treatment modalities and expertise availability etc.

## Consent

Written, informed consent was sought and attained from the father of the patient as per medical protocol in Nepal.
