# Supplementary material for: Case Report: Microsurgical excision of grade 5 cerebral AVM
Source: F1000Res. 2016 Jul 4;4:1197. Originally published 2015 Nov 2. [Version 2] doi: 10.12688/f1000research.7257.2 (PMC5089143; doi:10.12688/f1000research.7257.2)
Supplement: Peer review history

# Peer review: Referee response for version 2

**DOI:** 10.5256/f1000research.9835.r17066

**Published:** 2016-10-28

**Authors:** Guoyi Gao

**Affiliations:** 1 Department of Neurosurgery, Shanghai Jiao Tong University, Shanghai, China

This is a very successful case of surgical removal of high grade AVM which is not ideal for embolism. The experiences of decision making and surgical strategy are both beneficial to the readers.

I have read this submission. I believe that I have an appropriate level of expertise to confirm that it is of an acceptable scientific standard.

# Peer review: Referee response for version 2

**DOI:** 10.5256/f1000research.9835.r16467

**Published:** 2016-09-21

**Authors:** Sunil K Singh

**Affiliations:** 1 Department of Neurosurgery, King George's Medical University, Lucknow, India

I have seen the revised manuscript. I still see that the AVM appears overgraded as the size and eloquence appear doubtful, in absence of measurements.

I have read this submission. I believe that I have an appropriate level of expertise to confirm that it is of an acceptable scientific standard, however I have significant reservations, as outlined above.

# Peer review: Referee response for version 2

**DOI:** 10.5256/f1000research.9835.r15967

**Published:** 2016-08-31

**Authors:** Kenichiro Kikuta

**Affiliations:** 1 Department of Neurosurgery, University of Fukui, Fukui, Japan

This is an interesting paper but I do not agree that it was grade 5 AVM. The size is probably over 6cm, and deep drainage can also be confirmed, but it is unclear whether this AVM is located in an eloquent area or not.  I think the authors should answer this question before the article is indexed.

I have read this submission. I believe that I have an appropriate level of expertise to confirm that it is of an acceptable scientific standard, however I have significant reservations, as outlined above.

# Peer review: Referee response for version 1

**DOI:** 10.5256/f1000research.7819.r12755

**Published:** 2016-03-22

**Authors:** Sunil K Singh

**Affiliations:** 1 Department of Neurosurgery, King George's Medical University, Lucknow, India

It is a well written case report and the discussion is precise. Main points for not approving this article are:

1. Grading appears faulty as size of nidus, eloquence and drainage are suspicious on single images. more images might have been helpful. The AVM at best appears to be grade 3.
2. As such, it would be a routine microsurgical excision of an AVM

I have read this submission. I believe that I have an appropriate level of expertise to state that I do not consider it to be of an acceptable scientific standard, for reasons outlined above.

# Peer review: Referee response for version 1

**DOI:** 10.5256/f1000research.7819.r10999

**Published:** 2015-11-12

**Authors:** Kenichiro Kikuta

**Affiliations:** 1 Department of Neurosurgery, University of Fukui, Fukui, Japan

The authors reported a case of S-M grade 5 AVM successfully treated by microsurgical resection. I have some questions about as below.

1. How did the authors measure the size of AVM. How did they distinguish hematoma from AVM?  Was the AVM alone really above 6 cm in diameter? The authors should show the image such as T2-weighted images of MRI or enhanced CT clearly indicating the size of AVM.
2. Was the AVM really located in the eloquent area? The authors should show the location of nidus clearly. I think not nidus but only hematoma was located in the insult.
3. Magnification of  figures 3 and figures 4 was too high. The authors should show angiograms of  A-P view and lateral view with less magnification.
4. Why didn’t the authors perform preoperative embolization? I think it would be helpful in cases of high-grade AVMs. 

I have read this submission. I believe that I have an appropriate level of expertise to confirm that it is of an acceptable scientific standard, however I have significant reservations, as outlined above.
